# Effect of Lithium-Disilicate Liners on Bond Strength and Fracture Resistance of Bilayered Zirconia Systems: A Systematic Review of In Vitro Evidence

**DOI:** 10.3390/dj14010005

**Published:** 2025-12-22

**Authors:** Alexandra Cristina Maroiu, Magda Mihaela Luca, Anca Jivanescu

**Affiliations:** 1 Department of Prosthodontics, Faculty of Dental Medicine, “Victor Babes” University of Medicine and Pharmacy, 300070 Timisoara, Romania; maroiu.alexandra@umft.ro (A.C.M.); jivanescu.anca@umft.ro (A.J.); 2 Advanced and Digital Endodontic, Restorative and Prosthodontic Treatment (TADERP) Research Center, “Victor Babes” University of Medicine and Pharmacy, 300070 Timisoara, Romania; 3 Department of Pediatric Dentistry, Faculty of Dental Medicine, “Victor Babes” University of Medicine and Pharmacy, 300070 Timisoara, Romania

**Keywords:** zirconia, glass-ceramic liners, lithium compounds, dental bonding, shear strength

## Abstract

**Background**/**Objectives:** Chipping of the veneering part of the crown at the zirconia–porcelain interface remains a major complication of bilayered zirconia systems. This systematic review evaluated whether incorporating a lithium-disilicate (LD) liner or press-on or CAD-on interlayer between zirconia and veneer improves bond strength and fracture performance in vitro. **Methods:** Following PRISMA 2020, we searched PubMed, Scopus, and Web of Science up to 7 September 2025 for open-access English in vitro studies using LD-based interlayers at zirconia–veneer interfaces and reporting quantitative bond and/or fracture outcomes. Data included extracted materials, processing parameters, and mechanical results; due to heterogeneity, findings were synthesized descriptively and as the ratio-of-means (ROM). **Results:** Five in vitro studies from Korea, Thailand, and India met the criteria. LD interlayers increased microtensile bond strength from 18.83 to 44.20 MPa and from 19.6 to 47.7 MPa, and shear bond strength from 41.3 to 59.7 MPa, 11.40 ± 1.29 to 18.81 ± 1.76 MPa, and 21.5 to 60.2 MPa. Corresponding ROMs ranged from 1.46 to 2.80 (median 2.35), with thermocycled LD groups maintaining strengths >25 MPa. LD liners also raised crown fracture loads from ~2.18 to ~3.45 kN and characteristic strength from 3.42 to 5.64 kN, while chipping loads in implant crowns increased from ~0.34 to ~0.84 kN and global fracture from ~1.71 to ~1.93 kN. **Conclusions:** Across diverse zirconia–veneer configurations, LD interlayers consistently enhanced bond metrics and fracture/chipping resistance, supporting their use as a targeted interfacial strategy; however, clinical confirmation is still needed.

## 1. Introduction

Bilayered zirconia systems pair a high-toughness yttria-stabilized tetragonal zirconia polycrystal (Y-TZP) substructure with an esthetic porcelain veneer, but veneer chipping and interfacial debonding remain the dominant technical complications limiting wider indications. Clinical literature consistently reports higher veneer fracture and chipping rates for veneered zirconia than for metal–ceramic, monolithic zirconia, or glass-ceramic restorations. They identify the veneer/core interface as the principal weak link that either needs to be engineered and reinforced or bypassed by selecting fully monolithic designs [1,2,3]. Thermo-mechanical incompatibility and processing-induced residual thermal stresses in the veneering ceramic are key mechanistic contributors to chipping in veneered Y-TZP [4].

Lithium disilicate (LD) glass-ceramics comprise a silica-rich glass phase embedded with Li_2_Si_2_O_5_ crystallites, enabling hydrofluoric acid etching and silanization for strong glass–resin coupling while offering thermo-mechanical compatibility with common veneering porcelains [5]. Contemporary evidence reviews reaffirm hydrofluoric acid etching followed by a silane coupling agent as the most reliable adhesion strategy for silica-based ceramics such as LD [5,6]. When applied as a spray/slurry liner that is co-fired or pressed with the veneering ceramic, LD can create a graded, silica-containing interfacial layer that improves wetting and fosters elemental interdiffusion, while better matching coefficients of thermal expansion (CTE) between veneer and core to reduce cooling stresses [7,8]. Finite-element analysis and experimental work further suggest that minimizing CTE mismatch in veneered Y-TZP structures mitigates stress patterns associated with veneer chipping [4,9].

In this context, LD interlayers have been proposed as an interfacial engineering strategy. Microtensile and shear bond strength experiments indicate that introducing an LD liner or press-on or CAD-on LD veneer between zirconia and conventional veneering ceramics can markedly increase interfacial bond strength and alter failure modes [7,10,11]. A lithium disilicate spray-liner—prepared as a low-viscosity slurry of LD glass–ceramic and sprayed onto the zirconia surface before firing to form a thin glass–ceramic coating—has been shown to increase both microtensile bond strength and crown-level fracture loads versus no-liner controls, with improved reliability on Weibull analysis [7]. Other shear bond strength (SBS) studies have reported LD-based interfaces with bond strengths remaining above ISO 9693:2019 debonding thresholds after thermocycling [11,12]. Taken together, these in vitro signals suggest that the interfacial chemistry and thermal compatibility afforded by LD may translate into clinically meaningful reductions in veneer chipping.

Despite this, existing reviews on zirconia prostheses tend to address veneer chipping only at a high level or to survey a wide range of interfacial strategies (e.g., liner ceramics, surface treatments, modified cooling protocols) without isolating LD interlayers as a discrete intervention [1,2,3,13,14,15]. Consequently, there is limited consolidated evidence to answer a practical question faced by clinicians and technicians: Does placing a lithium disilicate liner, press-on, or CAD-on interlayer between zirconia and the veneering ceramic reliably improve bond strength and fracture/chipping resistance compared with conventional veneering or cemented LD crowns? A focused appraisal of LD interlayers is needed to determine whether their additional laboratory complexity and cost are justified by reproducible mechanical advantages.

Mechanistically, LD interlayers may (i) enhance veneer slurry wetting and mechanical interlocking through a smoother, silica-containing surface; (ii) provide a glass phase that couples chemically to veneering porcelains and resin cements; and (iii) harmonize CTEs between veneer and zirconia, redistributing residual tensile stresses that promote crack initiation [8,9]. Scanning electron microscopy and energy-dispersive spectroscopy have demonstrated compositional gradients and interdiffusion at zirconia–porcelain interfaces affected by liners and processing, lending plausibility to a graded, tougher interface when LD is used [8]. Integrating finite-element analysis insights, closer CTE matching is expected to temper tensile residual stresses in the veneer and reduce chip initiation [4,9]. Translationally, higher interfacial bond strength and greater fracture/chipping loads should mean fewer veneer defects—particularly for thin esthetic veneers—provided laboratory protocols (surface pretreatment, firing/pressing schedules, cooling rates, and fatigue aging) are standardized [4,5,6,7,8,9,15].

Therefore, this systematic review aimed to synthesize open-access in vitro evidence on lithium disilicate liners and press-on or CAD-on interlayers at zirconia–veneer interfaces, focusing on bond strength and fracture/chipping outcomes. We hypothesized that LD interlayers would (a) increase microtensile and shear bond strengths compared with non-LD or no-liner controls from the same experimental setup, and (b) improve fracture and chipping resistance of bilayered zirconia assemblies. By presenting ratio-of-means comparisons and summarizing key processing parameters, we sought to provide a quantitative yet clinically interpretable framework for deciding when LD interlayers merit consideration in veneered zirconia workflows.

## 2. Materials and Methods

### 2.1. Protocol and Registration

We followed the PRISMA 2020 guidance for reporting systematic reviews, as described by Page et al. [16]. PRISMA 2020 provides a checklist and flow diagram to enhance transparency in how studies are identified, selected, and synthesized; it does not itself define eligibility criteria but complements them by ensuring complete reporting (Supplementary Materials: Table S1). In our case, no meta-analysis was performed because the included in vitro studies differed substantially in zirconia and liner systems, thermocycling regimens, specimen geometries, and testing methods, precluding a statistically homogeneous quantitative synthesis. The aim was prospectively defined to include only open-access full-text experimental studies assessing LD liner/press-on layers at zirconia–veneer interfaces with bond and/or fracture outcomes. The study was prospectively registered with the Open Science Framework (OSF), which provides a time-stamped, publicly accessible archive of the protocol and planned methods to enhance transparency and reduce selective reporting.

### 2.2. Eligibility Criteria

Inclusion: (i) in vitro experimental studies; (ii) zirconia substrates (Y-TZP or partially stabilized variants) veneered via a lithium disilicate liner (spray/slurry/fusion glass) or an LD press-on or CAD-on layer that functions as an interlayer between zirconia and the veneering ceramic; (iii) quantitative outcomes including bond strength (shear or microtensile) and/or fracture/chipping strength of the bilayer; (iv) full-text, open-access English articles; and (v) at least one comparator group within the same study that did not receive an LD interlayer (e.g., no-liner, feldspathic layering, or cemented LD crown), such that the effect of the LD interface could be interpreted against a reference. Exclusion: clinical series; purely adhesive studies bonding LD or zirconia directly to tooth without an interlayer focus; paywalled or abstract-only reports; narrative reviews, letters, or technical notes; and zirconia-to-resin or LD-to-tooth protocols that did not address the zirconia–veneer interface. Only studies with full-text articles available in open-access format were included, because licensed content beyond our institutional access could not be reliably retrieved or extracted.

### 2.3. Information Sources and Search Strategy

Primary: PubMed/MEDLINE. Cross-checks: Scopus Web of Science for completeness (only open-access full texts kept). Example PubMed string (free-text + field tags): “(zirconia[Title/Abstract] OR yttria-stabilized[Title/Abstract] OR Y-TZP[Title/Abstract]) AND (lithium disilicate[Title/Abstract] OR lithium-silicate[Title/Abstract] OR “glass-ceramic liner”[Title/Abstract] OR press-on[Title/Abstract] OR “CAD-on”[Title/Abstract]) AND (bond strength[Title/Abstract] OR shear[Title/Abstract] OR microtensile[Title/Abstract] OR fracture[Title/Abstract] OR chipping[Title/Abstract). An English-language filter was applied because our review team lacked the resources to perform reliable translation and data extraction from non-English full-text articles. Candidate records were screened at the title/abstract level; full texts were verified for open-access status and for outcomes of interest. Studies were screened by duration, from inception to 7 September 2025.

**Figure 1:**
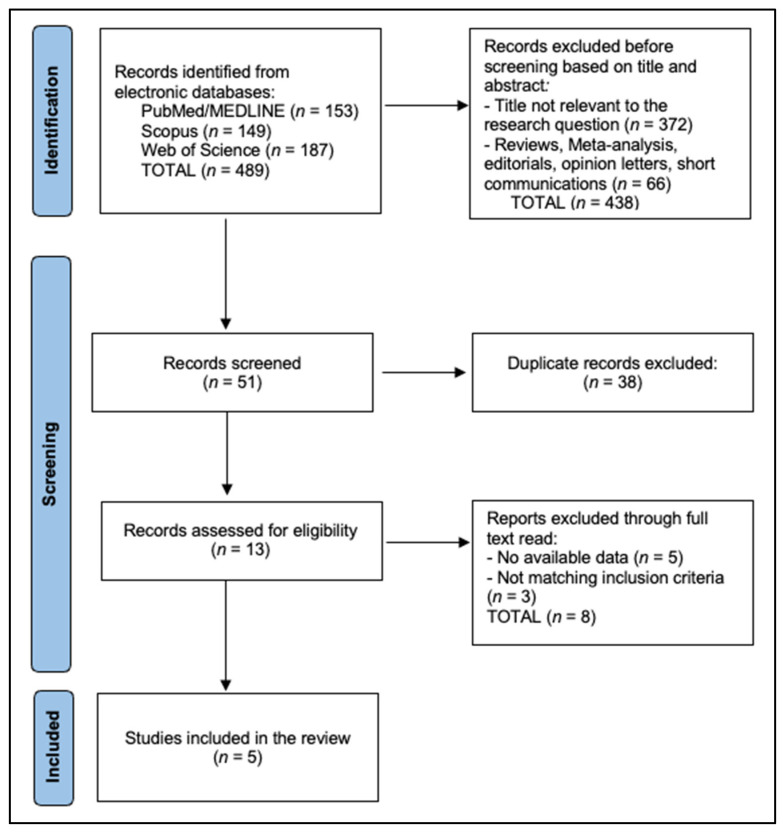
PRISMA Flowchart Diagram.

The PRISMA flowchart summarizes study selection across four phases. In the identification phase, 489 records were retrieved from electronic databases—PubMed/MEDLINE (*n* = 153), Scopus (*n* = 149), and Web of Science (*n* = 187). Before formal screening, 438 records were excluded based on titles and abstracts, comprising 372 that were not relevant to the research question and 66 secondary publications (reviews, meta-analyses, editorials, opinion letters, or short communications). This left 51 records for screening, during which 38 duplicates were removed. Consequently, 13 full-text articles were assessed for eligibility. Of these, eight were excluded after full-text review—five for having no available or extractable data and three for not meeting the predefined inclusion criteria. Ultimately, five studies met all criteria and were included in the review [17,18,19,20,21], as seen in Figure 1.

### 2.4. Study Selection and Data Extraction

Two reviewers independently screened and extracted data on zirconia/veneer materials, LD interlayer type (spray-liner vs. press-on or CAD-on), firing/press schedules, thermocycling/aging, test method (SBS, microtensile), statistics, and numerical outcomes (means, SD, Weibull, where available). Disagreements were resolved by consensus. When numeric values were not explicitly reported in the text or tables and could not be reliably extracted from figure images, we coded them as ‘NR’ (not reported) and interpreted the corresponding results qualitatively; we did not contact study authors to obtain additional data.

### 2.5. Risk of Bias

We qualitatively appraised the methodological rigor of the included in vitro studies across five prespecified domains commonly discussed in dental materials research: (i) sample size justification and random allocation of specimens to groups; (ii) blinding of operators and/or outcome assessors; (iii) presence and appropriateness of aging protocols (thermocycling and/or mechanical fatigue); (iv) completeness and transparency of reporting of materials (e.g., zirconia grade, liner and veneer brand), surface pretreatments, and firing/pressing schedules; and (v) appropriateness of statistical analysis (normality assessment, multiple-comparison adjustment, and reporting of dispersion measures). For each domain, we judged the risk of bias as ‘low’, ‘some concerns’, or ‘high’, and we then assigned an overall risk-of-bias judgment per study based on the worst domain. The results of this assessment are summarized in Table 1. We did not use a single validated risk-of-bias tool because, to date, there is no universally accepted instrument tailored to in vitro mechanical studies of prosthodontic materials; our approach was instead guided by methodological recommendations commonly applied in this literature, as presented in Table 1.

## 3. Results

Table 2 maps five open-access in vitro investigations spanning Korea, Thailand, and India and shows that all models explicitly inserted a lithium disilicate (LD) interlayer, either as a spray/liner co-fired with the zirconia or as a press-on or CAD-on veneer, to interrogate bond or fracture performance at the zirconia–veneer junction. Jang et al. [17] heat-treated a lithium disilicate reinforced spray liner on CAD/CAM 3Y-TZP at ~940–950 °C for 90 s before pressing, then measured microtensile bond strength and crown fracture reliability; no thermocycling was reported. Jang et al. [18] compared liner-bonding of a milled LD-reinforced crown to resin-cement bonding on zirconia abutments after 24 h water storage (no thermocycling), reporting microtensile bond, local chipping, and global fracture loads. Wattanasirmkit et al. [19] fired an LD glass–ceramic liner on zirconia prior to feldspathic veneering and performed 10,000 thermocycles before shear testing. Moses et al. [20] contrasted feldspathic layering versus heat-pressed LD over zirconia under 20,000 thermocycles, while Yadav et al. [21] layered a veneering porcelain (VITA VM9) over three interface variants, including a 0.5 mm IPS e.max Press liner fired at 930 °C, without thermocycling, and recorded shear bond strengths and SEM failure modes. Collectively, the table highlights consistent use of LD-based interfaces across diverse veneering routes and aging regimens, with primary outcomes centered on microtensile/shear bond strength and fracture/chipping resistance [17,18,19,20,21].

Table 3 summarizes bond strength outcomes and shows a uniform advantage for LD interfaces versus each study’s comparator. In microtensile testing, Jang et al. [17] reported 44.20 MPa with an LD spray-liner versus 18.83 MPa without a liner (Δ = +25.4 MPa), and Jang et al. [18] found 47.7 MPa for liner-bonded crowns versus 19.6 MPa for resin-cement-bonded crowns (Δ = +28.1 MPa). In shear testing, Wattanasirmkit et al. [19] achieved ~59.7 MPa pre-TC with values remaining >25 MPa after 10,000 cycles, exceeding commonly cited debonding thresholds. Moses et al. [20] showed significantly higher SBS for heat-pressed LD (18.81 ± 1.76 MPa) compared with feldspathic layering (11.40 ± 1.29 MPa; *p* < 0.01). Yadav et al. [21] listed the LD liner as the top-performing group versus SiO_2_-based and other interlayers, with adhesive failure predominance, though exact MPa values were not numerically reported in the table (NR). Overall, the compiled data indicate that LD liners or press-on schemes typically add ~7–28 MPa to controls and sustain strengths after aging, where applicable [17,18,19,20,21].

**Figure 2:**
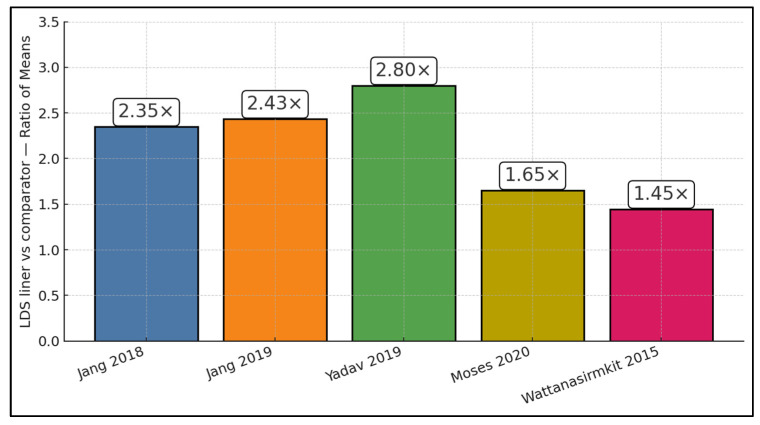
ROM per study [17,18,19,20,21].

Figure 2 compiles the study-level ratio of means for bond strength, showing consistently higher values with a lithium disilicate liner/press-on versus each study’s own comparator. The ROMs (LDS ÷ control) and raw means were: 2.35× in Jang et al. (μTBS 44.20 vs. 18.83 MPa) [17], 2.43× in Jang et al. (μTBS 47.70 vs. 19.60 MPa) [18], 2.80× in Yadav et al. 2019 (SBS 60.20 vs. 21.50 MPa) [21], 1.65× in Moses et al. 2020 (SBS 18.81 vs. 11.40 MPa) [20], and 1.46× in Wattanasirmkit et al. (SBS 59.70 vs. 41.30 MPa) [19]. The median ratio of means was 2.35× (IQR ≈ 1.65–2.43×), indicating that LD interfaces typically deliver ~65–143% higher bond strength than controls, with the largest relative gain observed in the Yadav SBS protocol [21].

Table 4 focuses on fracture and chipping behavior of bilayered zirconia assemblies and demonstrates that LD interfaces generally raise both local damage thresholds and global failure loads where quantified. Jang et al. [17] recorded a higher average load-to-fracture for LD-lined crowns (~3.45 kN) versus no-liner (~2.18 kN) and a substantially greater characteristic strength on Weibull analysis (5.64 versus 3.42 kN), indicating improved reliability. In an implant-crown model, Jang et al. [18] showed higher local chipping loads for liner-bonded crowns (~0.84 kN) than resin-cement-bonded (~0.34 kN), with a smaller yet favorable increase in global fracture load (~1.93 kN vs. ~1.71 kN). The remaining studies centered on bond testing and did not provide global fracture metrics (NR) [19,20,21]. Taken together, the available mechanical data suggest that, beyond boosting interfacial bond metrics, LD interlayers can shift failure from premature chipping toward higher-load, more catastrophic events, consistent with a tougher, more reliable interface [17,18,19,20,21].

Table 5 details processing variables that likely underpin performance differences across studies, emphasizing linear schedules, thermal/aging protocols, and test configurations. Jang et al. [18] uniquely quantifies interface thickness, reporting 33.6 ± 5.2 µm for the liner-bonded interface versus 13.3 ± 1.6 µm for the cement layer, alongside a crosshead speed of 0.5 mm/min and airborne abrasion (50 µm Al_2_O_3_, 3 bar) applied only to the cement-bonded group—an asymmetry that may influence failure modes. Wattanasirmkit et al. [19] used manufacturer press-on schedules and 20,000 thermocycles, while Moses et al. [20] varied firing temperature as an experimental factor with 5000–10,000 cycles and reported the highest SBS at 59.7 MPa under an LD-liner condition. Jang et al. [17] provided Weibull outputs for μTBS and crown fracture (μTBS σ_0_ rising from 20.57 to 49.38 MPa with liner), though several procedural specifics (crosshead speed, zirconia pretreatments) were NR. Yadav et al. [21] standardized a layered build-up (0.5 mm liner/dentin/enamel; total 4.5 mm) with firing at 930/910/900 °C and a crosshead speed of 0.5 mm/min. Overall, the table underscores heterogeneity in thermal histories and preparation steps—particularly thermocycling intensity and liner processing—that likely modulate both bond and fracture responses [17,18,19,20,21]. In the studies that reported Weibull analysis, the characteristic strength (σ_0_) represents the stress at which 63.2% of specimens are expected to fail, and the Weibull modulus (m) reflects the scatter of the failure distribution, with higher m indicating more reliable, less variable performance.

**Figure 3:**
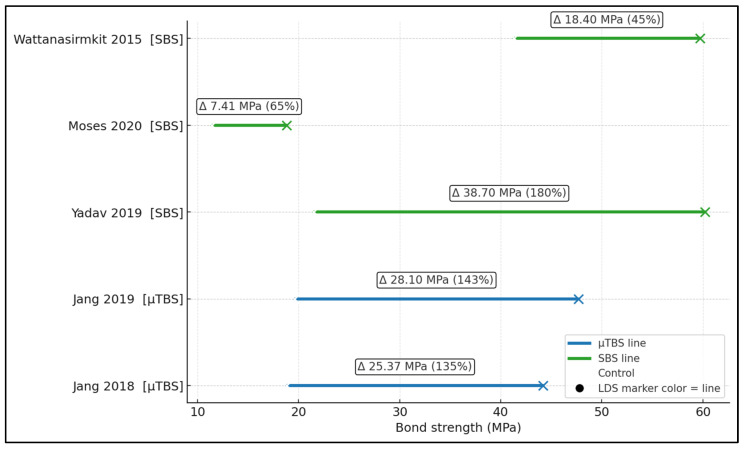
Dumbbell plot of absolute bond strengths (MPa) [17,18,19,20,21].

Figure 3 displays absolute mean bond strengths (MPa) as a dumbbell plot linking each study’s control to its LDS result, with the online annotation showing the mean difference and percent increase. Gains were +25.37 MPa (+135%) in Jang et al. [17], +28.10 MPa (+143%) in Jang et al. [18], +38.70 MPa (+180%) in Yadav et al. [21], +7.41 MPa (+65%) in Moses et al. [20], and +18.40 MPa (+45%) in Wattanasirmkit et al. [19]. Thus, while all models favored LDS, the largest absolute and percentage improvement occurred in the Yadav layered-zirconia SBS setup [21], whereas the smallest absolute gain occurred in the press-on vs. feldspathic comparison of Moses et al. [20]; μTBS studies still showed large absolute jumps (~25–28 MPa) with LDS liners [17,18].

## 4. Discussion

### 4.1. Summary of Evidence

Our findings that an LD interlayer raises zirconia–veneer bond strength and increases crown-level fracture resistance are consistent with foundational experimental work on residual thermal stresses in veneered Y-TZP. Using hole-drilling, Mainjot et al. showed that cooling history governs the residual stress profile in the veneer, helping to explain why interfaces that better accommodate cooling can resist chip initiation [22]. Extending that analysis, a later hole-drilling study from the same group demonstrated that minimizing the CTE mismatch (Δα) between veneer and Y-TZP produces more favorable stress distributions—supporting our rationale that a silica-rich LD interlayer, by grading the interface and harmonizing Δα, should reduce tensile residual stresses that promote chipping [23].

Across the five included studies, lithium disilicate interlayers consistently increased bond strength relative to each study’s internal control, with ratio-of-means values ranging from 1.46 to 2.80 and a median improvement of approximately 2.35-fold. In absolute terms, LD liners and press-on or CAD-on LD veneers added between roughly 7 and 28 MPa to non-LD interfaces, with the largest gains observed in microtensile bond strength designs and in the layered SBS setup of Yadav et al. [21]. Importantly, SBS values in thermocycled LD groups remained above the ISO 9693:2019 thresholds, suggesting that the LD interface can maintain functionally adequate adhesion even after aging cycles intended to simulate an oral environment [12,19,20].

Fracture and chipping data, although more limited, point in the same direction. Jang et al. [17] reported that an LD spray-liner increased both the mean and characteristic crown fracture loads by more than 1 kN compared with no-liner controls, indicating not only a higher average resistance but also improved reliability under Weibull analysis. In an implant-crown model, liner-bonded LD crowns exhibited more than double the local chipping load and a modest increase in global fracture load relative to resin-cement-bonded LD crowns, consistent with a tougher, more stable interface [18]. While these experiments do not replicate complex clinical loading, the fact that both interfacial bond metrics and crown-level failure loads shift in favor of LD interlayers—despite heterogeneous zirconia grades, liner formats, and aging regimens—strengthens the inference that the LD interface confers a genuine mechanical advantage.

Crown geometry and cooling protocols also modulate veneer stresses in ways that align with our data. In anatomically shaped zirconia molar crowns, Al-Amleh et al. found that veneer thickness and cooling rate significantly affected measured residual stresses, with thinner veneers under fast cooling developing higher surface compression—conditions that can be protective when interfacial compatibility is adequate [24]. Conversely, Göstemeyer et al. reported that “slow-cool” schedules, sometimes advocated for veneered zirconia, actually decreased interfacial adhesion energy in several zirconia/porcelain pairs, cautioning that slow cooling is not a universal solution and that Δα interactions matter—again pointing to the value of an LD interlayer that better matches thermo-mechanical behavior [25].

Beyond thermal compatibility, surface pretreatment choices interact with interlayer strategies. A 2024 MDPI study showed that grit-blasting sintered zirconia with 30-µm silica-coated alumina improved µTBS of a pressable veneering ceramic compared with pre-sinter blasting, with predominantly mixed failures—suggesting that micromechanical/chemical activation of the zirconia surface can complement a glass-containing interface [26]. Cheng et al. similarly observed that carefully selected alumina particle size/pressure (e.g., 50 µm at 0.3 MPa) enhances bond strength of heat-pressed veneers to zirconia and that thermocycling had negligible adverse effects under optimal parameters—consistent with our durability signal when LD is used as the pressed or fused interlayer [27].

The processing route also influences outcomes. Ishibe et al. reported comparable shear bond strengths for pressed versus layered porcelains on zirconia when systems were matched, highlighting that veneer processing per se is not the only determinant of adhesion; the material pairing and the interfacial chemistry are pivotal [28]. Still, at the crown level, CAD-on designs employing a lithium disilicate glass–ceramic over zirconia yielded higher fracture and cyclic-fatigue resistance than hand-layered veneers in an in vitro study, mirroring our observation that LD-based interlayers can translate into superior structural performance under load [29].

Failure analysis in the literature also fits our fracture-mode trends. Work comparing different zirconia surface treatments and liners (including laser and liner combinations) demonstrated that appropriately conditioned zirconia surfaces increase shear bond strength to veneering ceramics and can shift failures away from purely adhesive modes at the interface—compatible with our higher µTBS and mixed-failure patterns when an LD interlayer is used [30].

Finally, recent ISO-guided mechanical testing provides a benchmark for clinical acceptability. Dimitriadis et al. evaluated zirconia–porcelain bonds by ISO 9693 three-point bending and reported mean strengths of ≈32–34 MPa, well above the 20 MPa minimum, together with robust Weibull moduli, underscoring that interfaces meeting contemporary standards can deliver reliable performance [31]. Our SBS/µTBS gains with an LD interlayer align with (and in many cases exceed) these thresholds, strengthening the translational case for LD liners/press-on layers as a pragmatic route to mitigate chipping in bilayered zirconia systems.

When veneering zirconia for esthetic demands, incorporating an LD interlayer—either as a heat-bonded liner prior to porcelain build-up or as a press-on or CAD-on LD veneer—appears to meaningfully increase interfacial bond strength (often by ~20–30 MPa) and elevate chipping/fracture loads. Practically, this favors LD-based bilayers in situations with thin veneering ceramics or high occlusal load, provided the lab adheres to controlled firing/press schedules, maintains conservative cooling to limit residual stresses, and standardizes surface treatments. Where screw-retained implant crowns risk veneer chipping, liner-bonding to zirconia, clinicians may consider LD-based interlayers as a promising option in cases where esthetics necessitate veneering rather than fully monolithic restorations, particularly for thin veneering ceramics or high occlusal loads. However, such protocols should be implemented in close collaboration with laboratories and interpreted cautiously until supported by prospective clinical data.

Nevertheless, our synthesis does not allow a definitive ranking of the relative importance of interfacial composition versus processing and aging; several patterns are nevertheless noteworthy. First, the magnitude and consistency of the bond strength gains associated with lithium disilicate interlayers—often on the order of 20–30 MPa—suggest that the chemical and thermo-mechanical compatibility of the LD interface (including its silica content and CTE) is a primary driver of improved adhesion. Second, studies that combined LD interlayers with thermocycling [19,20] still reported strengths above ISO thresholds, indicating that the LD interface retained its advantage under mechanical and thermal stress. At the same time, differences in layering versus press-on techniques and in cooling rates clearly modulated absolute values [17,18,19,20,21]. Overall, our data support the view that the interfacial ingredients (lithium disilicate glass–ceramic) provide the underlying potential for stronger, more reliable bonding. However, this potential can be enhanced or undermined by the way the veneer is layered or pressed, as well as by the applied aging protocol.

### 4.2. Limitations

The evidence base is limited to in vitro studies with small samples, heterogeneous zirconia grades, liner products, firing/pressing schedules, and aging protocols. Thermocycling and mechanical fatigue regimens varied and were sometimes incompletely reported; some reports lacked numeric tabulation outside figures. In addition, we restricted inclusion to English-language, open-access full-text articles because licensed content beyond our institutional access could not be consistently retrieved or extracted. This language and access filter may have excluded 20–30% of potentially relevant studies, introducing a risk of selection bias. Testing geometries (SBS vs. μTBS, chisel vs. notched) and asymmetries in surface pretreatments further complicate cross-study comparison and preclude formal meta-analysis. Publication bias cannot be excluded, and in vitro gains may overestimate clinical benefit due to simplified loading and environmental conditions.

## 5. Conclusions

Open-access bench data indicate that lithium disilicate interlayers at the zirconia–veneer interface consistently strengthen bonding and improve fracture/chipping performance versus conventional veneering or cemented LD crowns. While clinical confirmation is still needed, adopting LD liner, press-on, or CAD-on strategies—with meticulous laboratory execution—offers a rational pathway to mitigate the well-recognized veneer/core interface vulnerability in veneered zirconia restorations.

## Figures and Tables

**Table 1:** Qualitative risk-of-bias assessment of the included in vitro studies.

Study	Randomization and Allocation	Aging Protocol (Thermocycling/Fatigue)	Reporting of Materials and Protocols	Statistics (Analysis and Reporting)	Overall Risk of Bias
Jang et al. 2018 [17]	NR; groups defined, but no randomization description	Water storage only; no thermocycling → some concerns	Zirconia and liner materials described; firing schedule partially specified → some concerns	Appropriate group comparisons; dispersion reported; no power calculation → some concerns	High (limited aging)
Jang et al. 2019 [18]	NR; allocation method not stated	Water storage only; no thermocycling → high concerns for long-term behavior	Materials and bonding protocols described; interface thickness reported → low	Appropriate statistics; no power calculation → some concerns	High
Wattanasirmkit et al. 2015 [19]	NR	Thermocycling (10,000 cycles) reported → low	Materials, liner, and press-on protocols described → low	Appropriate tests; dispersion reported; no power calculation → some concerns	Some concerns
Moses et al. 2020 [20]	NR	Thermocycling (5000–10,000 cycles) as experimental factor → low	Liner types, firing temperatures, and cycles described → low	Three-way ANOVA with post hoc tests; no power calculation → some concerns	Some concerns
Yadav et al. 2019 [21]	NR	Thermocycling n.r.; only storage → some concerns	Materials, liner thickness, and firing temperatures detailed → low	Appropriate statistical comparisons; dispersion reported; no power calculation → some concerns	Some concerns

NR—Not Reported.

**Table 2:** Characteristics of included studies.

Study (Year; Journal)	Country/Setting	Zirconia and Veneer Configuration	LD Interface Type (Brand/Processing)	Thermocycling/Aging	Primary Outcomes
Jang et al. 2018 [17]	Korea (in vitro)	CAD/CAM 3Y-TZP zirconia core + heat-pressed lithium-disilicate glass–ceramic veneer (Amber LiSi-POZ)	Lithium disilicate–reinforced spray-type liner (Hass, Seoul, Republic of Korea) applied to zirconia; heated to ~940–950 °C for 90 s before heat-press veneering	Water storage 24 h at 37 °C (no thermocycling reported)	Microtensile bond strength; crown fracture strength (with Weibull analysis)
Jang et al. 2019 [18]	Korea (in vitro)	Implant-supported CAD/CAM zirconia abutment (3Y-TZP) + milled lithium-disilicate–reinforced glass–ceramic crown (Amber Mill-Q)	Liner bonding (heat-bonding) of the LD-reinforced ceramic to zirconia versus dual-cure self-adhesive resin cement (cement-bonding)	Water storage 24 h at 37 °C prior to testing (no thermocycling reported)	Microtensile bond strength; initial chipping load; fracture resistance of implant-supported crowns
Wattanasirmkit et al. 2015 [19]	Thailand (in vitro)	Zirconia substructure + veneering porcelain	Lithium disilicate glass–ceramic liner fired on zirconia prior to veneering (vs no-liner)	Thermocycling: 10,000 cycles (protocol present in full text; abstract confirms 10 k cycles; detailed temps/dwell not stated in abstract)	Shear bond strength (SBS); phase/microstructure
Moses et al. 2020 [20]	India (in vitro)	Zirconia core + veneering by (A) feldspathic layering vs. (B) heat-pressed lithium disilicate over zirconia	Press-on LD scheme (vs feldspathic layering); no separate liner arm	Thermocycling: 20,000 cycles (per methods in open-access article)	Shear bond strength (SBS)
Yadav et al. 2019 [21]	India (in vitro)	VITA 3Y-TZP disks veneered with VITA VM9 dentin/enamel	Three interface variants vs. control: (1) Lithium disilicate glass–ceramic liner (IPS e.max Press) fired at 930 °C (0.5 mm), (2) Silicon-dioxide-based liner (VITA), (3) Glass-ceramic interlayer; each followed by VM9 dentin (910 °C) and enamel (900 °C)	No thermocycling stated; specimens finished to uniform thickness; universal testing without prior TC	SBS; SEM failure mode

3Y-TZP = 3-mol% yttria-stabilized tetragonal zirconia polycrystal; LD = lithium disilicate; VM9 = VITA veneering porcelain; SEM = scanning electron microscopy; TC = thermocycling.

**Table 3:** Bond strength outcomes (microtensile/SBS).

Study	Test	Group(s) Compared	Bond Strength (Mean ± SD)	Δ vs. Control/Notes
Jang et al. 2018 [17]	Microtensile	LD spray-liner vs. no-liner	44.20 MPa (liner) vs. 18.83 MPa (no-liner)	Liner +25.4 MPa
Jang et al. 2019 [18]	Microtensile	Liner-bonded vs. resin-cement-bonded	47.7 MPa (liner-bonded) vs. 19.6 MPa (cement-bonded)	Liner +28.1 MPa
Wattanasirmkit et al. 2015 [19]	Shear (SBS)	LD glass–ceramic liner (best condition)	~59.7 MPa (pre-TC); >25 MPa post-10,000 TC	Highest SBS observed; remained > ISO threshold
Moses et al. 2020 [20]	Shear (SBS)	LD press-on vs. feldspathic layering	18.81 ± 1.76 MPa (LD) vs. 11.40 ± 1.29 MPa (feldspathic)	LD significantly higher (*p* < 0.01)
Yadav et al. 2019 [21]	Shear (SBS)	LD liner vs. SiO_2_ liner vs. glass–ceramic interlayer vs. control	NR (LD highest per text; numeric in image-only table)	LD ranked highest; failure mostly adhesive

SBS = shear bond strength; LD = lithium disilicate; TC = thermocycling; NR = not reported; ISO = International Organization for Standardization.

**Table 4:** Fracture/chipping performance of bilayered zirconia assemblies.

Study	Specimen/Test	Groups	Chipping/Local Fracture	Global Fracture (Weibull Mean or Mean)	Notes
Jang et al. 2018 [17]	Crown-like; load-to-fracture; Weibull	LD spray-liner vs. no-liner	—	σf(avg): ~3.45 kN (liner) vs. ~2.18 kN (no-liner); characteristic strength 5.64 vs. 3.42 kN	Liner increased both average and characteristic fracture strengths
Jang et al. 2019 [18]	Implant crown; local chipping and global fracture	Liner-bonded vs. resin-cement-bonded	Chipping: ~0.84 kN (liner) vs. ~0.34 kN (cement)	Fracture: ~1.93 kN (liner) vs. ~1.71 kN (cement)	Liner improved both chipping resistance and overall fracture load
Wattanasirmkit et al. 2015 [19]	Bilayer blocks; SBS focus	LD press-on vs. feldspathic	—	NR	Study centered on SBS; fracture not quantified
Moses et al. 2020 [20]	SBS focus, with microstructure	LD liner vs. no-liner	—	NR	Fractography reported (mixed failures); no global fracture load
Yadav et al. 2019 [21]	SBS + failure mode	LD liner vs. others	Adhesive failure dominant with LD	NR	No load-to-fracture data

kN = kilonewton; NR = not reported.

**Table 5:** Preparation/testing.

Study (Year)	Liner/Product	Liner Firing Protocol (°C)	Veneer/Crown Method	thermocycling (Cycles)	Crosshead Speed (mm/min)	Zirconia Pretreatment	Interface Thickness (µm)	Additional Findings
Jang et al. 2018 [17]	Lithium disilicate “liner” forming chemical interlayer	NR (liner heat-bonded during build-up)	Layered porcelain on zirconia	NR	NR	NR	NR	Weibull (μTBS): m = 2.79 (no-liner) vs. 4.77 (liner); σ_0_ (μTBS): 20.57 vs. 49.38 MPa; crown–core characteristic fracture load σ_0_: 5.64 kN (liner) vs. 3.42 kN.
Jang et al. 2019 [18]	Li-disilicate-reinforced glass–ceramic liner (Amber Mill-Q liner)	~800 °C heat-bonding (manufacturer schedule)	CAD/CAM glass–ceramic crown, liner-bonded vs. resin-cement-bonded	NR	0.5	Airborne abrasion (50 µm Al_2_O_3_, 3 bar) in the cement-bonded group only	33.6 ± 5.2 (liner) vs. 13.3 ± 1.6 (cement layer)	Initial chipping: 843.8 ± 317.5 N (liner) vs. 341.0 ± 90.2 N; fracture: 1929.6 ± 191.1 N (liner) vs. 1711.1 ± 275.4 N (ns).
Wattanasirmkit et al. 2015 [19]	IPS e.max Press-on over Y-TZP vs. feldspathic layering	Manufacturer schedules; press-on cycle for e.max	Press-on vs. conventional layering	20,000	NR	NR	NR	Reported significantly higher SBS for press-on Li-disilicate vs. feldspathic and polymer-infiltrated ceramic after 20 k cycles
Moses et al. 2020 [20]	Li-disilicate glass–ceramic liner (experimental) vs. other liners	Firing temperature significantly affected SBS (exact °C combinations per protocol)	Layered feldspathic porcelain on Zr	5000–10,000 (factor in three-way ANOVA)	NR	NR	NR	Highest SBS reported: 59.7 MPa (Li-disilicate liner condition).
Yadav et al. 2019 [21]	0.5 mm Li-disilicate liner (IPS e.max Press) vs. SiO_2_-based liner, glass–ceramic interlayer, control	930 °C (liner); dentin 910 °C; enamel 900 °C	Layered (0.5 mm liner + 0.5 mm dentin + 0.5 mm enamel; total 4.5 mm)	NR	0.5	NR	NR	Outcome direction: The Li-disilicate liner showed the highest mean SBS among groups; detailed MPa values are provided in the figure/table images.

Al_2_O_3_ = aluminum oxide; μm = micrometer; μTBS = microtensile bond strength; SBS = shear bond strength; NR = not reported; Weibull ‘characteristic strength’ (σ_0_) = scale parameter indicating the stress at which 63.2% of specimens are expected to fail; m = Weibull modulus (shape parameter), where higher m denotes lower variability and greater reliability.

## Data Availability

No new data were created or analyzed in this study. Data sharing is not applicable to this article.

## Supplementary Materials

The following supporting information can be downloaded at: https://www.mdpi.com/article/10.3390/dj14010005/s1, Table S1: PRISMA 2020 Checklist.

## References

[1] Agustín-Panadero R., Román-Rodríguez J.L., Ferreiroa A., Solá-Ruíz M.F., Fons-Font A. (2014). Zirconia in fixed prosthesis. A literature review. J. Clin. Exp. Dent..

[2] Raigrodski A.J., Hillstead M.B., Meng G.K., Chung K.H. (2012). Survival and complications of zirconia-based fixed dental prostheses: A systematic review. J. Prosthet. Dent..

[3] Pjetursson B.E., Valente N.A., Strasding M., Zwahlen M., Liu S., Sailer I. (2018). A systematic review of the survival and complication rates of zirconia-ceramic and metal-ceramic single crowns. Clin. Oral Implant. Res..

[4] Tanaka C.B., Ballester R.Y., De Souza G.M., Zhang Y., Meira J.B. (2019). Influence of residual thermal stresses on the edge chipping resistance of PFM and veneered zirconia structures: Experimental and FEA study. Dent. Mater..

[5] Zhang Y., Vardhaman S., Rodrigues C.S., Lawn B.R. (2023). A Critical Review of Dental Lithia-Based Glass-Ceramics. J. Dent. Res..

[6] Moreira P.M., Carvalho G.L.M., de Castro Albuquerque R., André C.B. (2024). Effect of hydrofluoric acid and self-etch ceramic primers on the flexural strength and fatigue resistance of glass ceramics: A systematic review and meta-analysis of in vitro studies. Jpn. Dent. Sci. Rev..

[7] Aguilera M.A.R., Bortolazzo A.C., Correr-Sobrinho L., Consani R.L.X. (2024). Surface treatments of the zirconia-reinforced lith-ium disilicate ceramic in the adhesion to the resin cement. Braz Dent J..

[8] Alghazzawi T.F., Janowski G.M. (2016). Effect of liner and porcelain application on zirconia surface structure and composition. Int. J. Oral Sci..

[9] Jikihara A.N., Tanaka C.B., Ballester R.Y., Swain M.V., Versluis A., Meira J.B. (2019). Why a zero CTE mismatch may be better for veneered Y-TZP structures. J. Mech. Behav. Biomed. Mater..

[10] Renda J.J., Harding A.B., Bailey C.W., Guillory V.L., Vandewalle K.S. (2015). Microtensile bond strength of lithium disilicate to zirconia with the CAD-on technique. J. Prosthodont..

[11] Kim S.H., Park C.J., Cho L.R., Huh Y.H. (2018). Evaluation of the ceramic liner bonding effect between zirconia and lithium disilicate. J. Prosthet. Dent..

[12] (2019). Dentistry—Compatibility Testing for Metal- and Ceramic-Ceramic Systems.

[13] Sreekala L., Narayanan M., Eerali S.M., Eerali S.M., Varghese J., Zainaba Fathima A.L. (2015). Comparative evaluation of shear bond strengths of veneering porcelain to base metal alloy and zirconia substructures before and after aging—An in vitro study. J. Int. Soc. Prev. Community Dent..

[14] Siarampi E., Sarafidou K., Papadopoulou L., Kantiranis N., Kontonasaki E., Koidis P. (2022). Effect of different zirconia surface pretreatments on the flexural strength of veneered Y-TZP ceramic before and after in vitro aging. J. Prosthodont. Res..

[15] Fischer J., Stawarzcyk B., Trottmann A., Hämmerle C.H. (2009). Impact of thermal misfit on shear strength of veneering ceramic/zirconia composites. Dent. Mater..

[16] Page M.J., McKenzie J.E., Bossuyt P.M., Boutron I., Hoffmann T.C., Mulrow C.D., Shamseer L., Tetzlaff J.M., Akl E.A., Brennan S.E. (2021). The PRISMA 2020 statement: An updated guideline for reporting systematic reviews. BMJ.

[17] Jang Y.-S., Noh H.-R., Lee M.-H., Lim M.-J., Bae T.-S. (2018). Effect of Lithium Disilicate Reinforced Liner Treatment on Bond and Fracture Strengths of Bilayered Zirconia All-Ceramic Crown. Materials.

[18] Jang Y.-S., Oh S.-H., Oh W.-S., Lee M.-H., Lee J.-J., Bae T.-S. (2019). Effects of Liner-Bonding of Implant-Supported Glass–Ceramic Crown to Zirconia Abutment on Bond Strength and Fracture Resistance. Materials.

[19] Wattanasirmkit K., Srimaneepong V., Kanchanatawewat K., Monmaturapoj N., Thunyakitpisal P., Jinawath S. (2015). Improving shear bond strength between feldspathic porcelain and zirconia substructure with lithium disilicate glass-ceramic liner. Dent. Mater. J..

[20] Moses A., Ganesan L., Shankar S., Hariharan A. (2020). A comparative evaluation of shear bond strength between feldspathic porcelain and lithium di silicate ceramic layered to a zirconia core—An in vitro study. J. Clin. Exp. Dent..

[21] Yadav P., Dabas N., Phukela S.S., Malhotra P., Drall S., Ritwal P.K. (2019). A comparative evaluation of the effect of liners on the shear bond strength of veneered zirconia block: An in vitro study. J. Indian. Prosthodont. Soc..

[22] Mainjot A.K., Schajer G.S., Vanheusden A.J., Sadoun M.J. (2011). Influence of cooling rate on residual stress profile in veneering ceramic: Measurement by hole-drilling. Dent. Mater..

[23] Mainjot A.K., Najjar A., Jakubowicz-Kohen B.D., Sadoun M.J. (2015). Influence of thermal expansion mismatch on residual stress profile in veneering ceramic layered on zirconia: Measurement by hole-drilling. Dent. Mater..

[24] Al-Amleh B., Neil Waddell J., Lyons K., Swain M.V. (2014). Influence of veneering porcelain thickness and cooling rate on residual stresses in zirconia molar crowns. Dent. Mater..

[25] Göstemeyer G., Jendras M., Dittmer M.P., Bach F.-W., Stiesch M., Kohorst P. (2010). Influence of cooling rate on zirconia/veneer interfacial adhesion. Acta Biomater..

[26] Zicari F., Monaco C., Vivan Cardoso M., Silvestri D., Van Meerbeek B. (2024). Bonding Effectiveness of Veneering Ceramic to Zirconia after Different Grit-Blasting Treatments. Dent. J..

[27] Cheng C.W., Yang C.C., Yan M. (2018). Bond strength of heat-pressed veneer ceramics to zirconia with various blasting conditions. J. Dent. Sci..

[28] Ishibe M., Raigrodski A.J., Flinn B.D., Chung K.H., Spiekerman C., Winter R.R. (2011). Shear bond strengths of pressed and layered veneering ceramics to high-noble alloy and zirconia cores. J. Prosthet. Dent..

[29] Pandurangan K.K., Veeraiyan D.N., Nesappan T. (2020). In vitro evaluation of fracture resistance and cyclic fatigue resistance of computer-aided design-on and hand-layered zirconia crowns following cementation on epoxy dies. J. Indian. Prosthodont. Soc..

[30] Kirmali O., Akin H., Ozdemir A.K. (2013). Shear bond strength of veneering ceramic to zirconia core after different surface treatments. Photomed. Laser Surg..

[31] Dimitriadis K., Tulyaganov D.U., Agathopoulos S. (2024). Evaluation of bond strength between zirconia milled ceramic material and veneered dental porcelain. Eur. J. Oral Sci..

